# Other bricks for the correct construction of the mitochondrial permeability transition pore complex

**DOI:** 10.1038/cddis.2017.96

**Published:** 2017-03-23

**Authors:** Giampaolo Morciano, Massimo Bonora, Carlotta Giorgi, Paolo Pinton

**Affiliations:** 1 Department of Morphology, Surgery and Experimental Medicine, Section of Pathology, Oncology and Experimental Biology, Laboratory for Technologies of Advanced Therapies (LTTA), University of Ferrara, Ferrara, Italy

In recent years, the concept of the mitochondrial permeability transition pore (mPTP) has attracted considerable attention from researchers in the field of pathophysiology, focusing on mitochondrial function as a potential therapeutic target. Indeed, given that the mPTP is considered to be the main and final effector of cell death in various disorders,^1, 2^ numerous and progressive efforts have been undertaken using this novel molecular target, both in clinical trials^3, 4^ and especially in basic research. Although the exact structure of this supramolecular entity is largely unknown, studies beginning in 2013 have begun to elucidate the structure of the pore-forming component – the C subunit of the F_1_/F_O_ ATP synthase.^5, 6, 7, 8^

In 2013, two independent groups described that the potential role of the C subunit in pore opening depends on its expression levels^7^ and its phosphorylation status.^6^ Then, Alavian *et al.*^5^ demonstrated that the purified C subunit, when reconstituted into liposomes forms a voltage-sensitive channel, leading to the rapid and uncontrolled loss of the membrane permeability transition (MPT).

These studies contributed to the hypothesis that F_1_/F_O_ ATP synthase C subunit (possibly in its c-ring form) generates a nonspecific pore on the inner mitochondrial membrane that is responsible for the permeability transition under precise conditions. Nonetheless, such a hypothesis lacks a mechanistic explanation as to how a high-stability, lipid-filled c-ring could exit the dimeric F_1_/F_O_ ATP synthase complex and undergo a marked rearrangement that would allow for channel formation.^9^

A recent article from Pavlov *et al.* published in *Cell Death Discovery* provides a new insight into this, as yet poorly understood mechanism of mPTP formation. In this work, Elustondo *et al.*^10^ provide an elegant confirmation of previous reports and better define the mechanism of action of this crucial MPT event. In their study, they utilized sophisticated biochemical methods and a standardized *in vivo* approach to provide a strong link between calcium-induced mPTP, *de novo* assembly of the channel comprising the C subunit, and tissue damage in a model of ischemia–reperfusion injury in the brain.^10^ These authors advanced the possibility that mPTP channel assembly might be stimulated by a mitochondrial calcium trigger signal, similar to mPTP channel opening as described in their previous studies.^11^

First, they show that a certain amount of C subunit could be chloroform-extracted from mitochondria with induced MPT, whereas a negligible amount was obtained when the mPTP was not stimulated or inhibited. This event was independent of its expression levels, suggesting that the C subunit changes its interaction with dimeric F_1_/F_O_ ATP synthase, resulting in MPT.^10^

In a previous publication, the same group reported that a voltage-dependent channel, including polyhydroxybutyrate (PHB) and inorganic polyphosphate (polyP), could be isolated by chloroform extraction from mitochondria. It has already been reported that polyP is required for mPTP opening in different cell lines^12^ and that PHB localizes to mitochondria where it induces PTP.^13^ Indeed, in this paper, they were able to reaffirm that PHB and polyP were collected from mitochondria with a calcium-triggered mPTP, and that their levels correlated with C subunit levels.

It is widely recognized that the mPTP molecular pathway is deeply involved in ischemia–reperfusion injuries, such as myocardial infarction,^8, 14, 15^ stroke, and liver and kidney transplantation. By studying a model of *in vivo* mPTP-dependent stroke damage, the authors demonstrate high C subunit accumulation in the injured hemisphere, as compared with control hemisphere, convincingly substantiating the function and requirement of the C subunit/polyP/PHB triad in a translational context.

As proposed by the authors, during Ca^2+^-induced MPT, the C subunit associates with polyP and PHB, promoting the generation of a water-permeable channel.^10^ Given that the C subunit is a hydrophobic protein with properties very similar to those of lipids, it is not expected to be able to form water-filled pores in its c-ring form (Figure 1). These data suggest that the C subunit is responsible for forming the calcium-dependent channel with the help of polyP possibly serving as the hydrophilic coating of the pore.

Mechanistically, this work leaves questions regarding how the C subunit exits the ATP synthase complex: are some major rearrangements required? Is the process catalyzed by other known mPTP components or by polyP or PHB themselves? Nonetheless, these study takes great strides toward a more complete understanding of MPT. Alongside parallel discoveries of other new and important modulators, this work further supports the C subunit-centric vision of mPTP. We are confident that further studies will begin to explore and describe the use of this target in counteracting ischemia–reperfusion injury-based diseases.

## Figures and Tables

**Figure 1:**
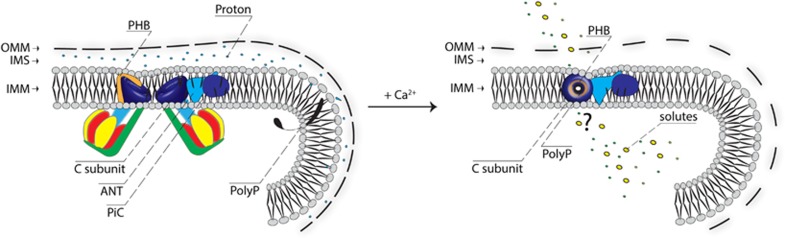
Hypothetical structure of the central conducting pore part. A portion of the mitochondrial structure is represented. In healthy cells (on the left), C subunit is part of the dimeric F_1_/F_O_ ATP synthase and contributes to ATP production (in blue – the C-ring). The model proposed by the authors (on the right) is that during Ca^2+^-induced MPT, the C subunit associates with polyP (in black) and PHB (in orange) allowing the generation of a water-permeable channel. A question mark has been inserted in the figure because other rearrangements could be required
